# Bioinspired 3D flexible devices and functional systems

**DOI:** 10.1093/nsr/nwad314

**Published:** 2023-12-13

**Authors:** Xu Cheng, Zhangming Shen, Yihui Zhang

**Affiliations:** Applied Mechanics Laboratory, Department of Engineering Mechanics, Tsinghua University, Beijing 100084, China; Laboratory of Flexible Electronics Technology, Tsinghua University, Beijing 100084, China; Applied Mechanics Laboratory, Department of Engineering Mechanics, Tsinghua University, Beijing 100084, China; Laboratory of Flexible Electronics Technology, Tsinghua University, Beijing 100084, China; Applied Mechanics Laboratory, Department of Engineering Mechanics, Tsinghua University, Beijing 100084, China; Laboratory of Flexible Electronics Technology, Tsinghua University, Beijing 100084, China

**Keywords:** bioinspired designs, biomimetic 3D structures, 3D flexible electronics, 3D functional systems, direct 3D manufacture, 2D-to-3D assembly

## Abstract

Flexible devices and functional systems with elaborated three-dimensional (3D) architectures can endow better mechanical/electrical performances, more design freedom, and unique functionalities, when compared to their two-dimensional (2D) counterparts. Such 3D flexible devices/systems are rapidly evolving in three primary directions, including the miniaturization, the increasingly merged physical/artificial intelligence and the enhanced adaptability and capabilities of heterogeneous integration. Intractable challenges exist in this emerging research area, such as relatively poor controllability in the locomotion of soft robotic systems, mismatch of bioelectronic interfaces, and signal coupling in multi-parameter sensing. By virtue of long-time–optimized materials, structures and processes, natural organisms provide rich sources of inspiration to address these challenges, enabling the design and manufacture of many bioinspired 3D flexible devices/systems. In this Review, we focus on bioinspired 3D flexible devices and functional systems, and summarize their representative design concepts, manufacturing methods, principles of structure-function relationship and broad-ranging applications. Discussions on existing challenges, potential solutions and future opportunities are also provided to usher in further research efforts toward realizing bioinspired 3D flexible devices/systems with precisely programmed shapes, enhanced mechanical/electrical performances, and high-level physical/artificial intelligence.

## Teaser

This review provides an overview of the latest progress in bioinspired 3D flexible devices and functional systems, highlighting design concepts, manufacturing methods, structure-function principles, applications, and future directions in novel 3D flexible devices with precisely programmed shapes, enhanced mechanical/electrical performances and high-level physical/artificial intelligence.

## INTRODUCTION

A prevailing trend in the development of flexible devices and functional systems involves the evolution of traditional planar layouts of solid-state elements into elaborated three-dimensional (3D) architectures, which has attracted intense attention from a variety of engineering fields [1–5]. Owing to their capabilities in providing novel functionalities and unprecedented mechanical/electrical performances with optimized structural/material designs, 3D flexible devices and functional systems have been utilized in a broad range of application scenarios, such as high-efficiency energy harvesting [6,7], environmental perception [8–11], human-machine interactions [12–14], health monitoring [15–17] and drug delivery [18–20]. Driven by these practical needs, 3D flexible devices and functional systems are rapidly evolving along three primary directions: (i) miniaturization (e.g. with the feature size reduced from millimeter scale to sub-microscale and nanoscale); (ii) increasing degree of physical intelligence (e.g. high-sensitivity perceptions, multimodal locomotion and self-healing) and embedded artificial intelligence (e.g. self-learning, judging and decision making); and (iii) enhanced adaptability and capabilities of heterogenous integration (especially with biological tissues and organs). However, ongoing explorations of these directions have encountered intractable challenges. For example, autonomous locomotion of 3D soft robotics with tens of microns in body length is very difficult to realize, due to the strong adhesion induced by both van der Waals and capillary forces [21]. Synchronous perception of multiple physical signals (e.g. pressure, in-plane strains, high-frequency vibration and ambient temperature) is an innate ability for human skin, while it is very challenging to impart such biological merit to artificial 3D sensors because of the signal coupling and multipath crosstalk [22]. In addition, maintaining 3D conformal integration with dynamically growing cells/tissues/organs (especially for infants) represents a significant technical challenge, where undesired delamination, mechanical constraint or penetration could occur due to the mismatch between engineering and biological materials/structures [23].

**Figure 1.**
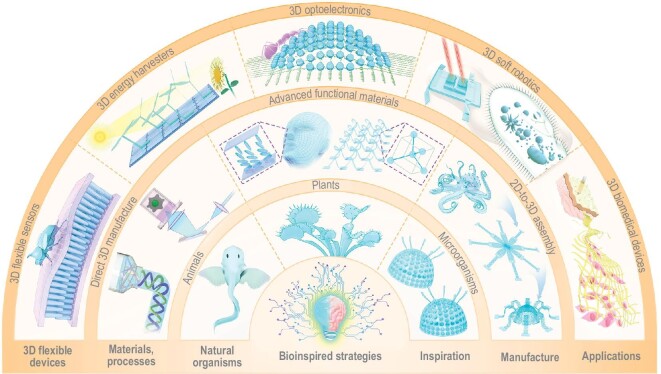
An overview of design strategies, manufacturing methods and representative applications of bioinspired 3D flexible devices. Inspired by the biological structures of natural organisms and principals of structure-function relation, a variety of 3D flexible devices/systems based on advanced materials and processing techniques have been reported, enabling many compelling applications, such as 3D flexible sensors [9], energy harvesters [49], optoelectronics [67], soft robotics [21] and biomedical devices [96].

By virtue of diverse, elaborately engineered and long-time–optimized processes, materials, structures and locomotion modes, nature has served scientists and engineers as a vital source of inspiration [24–27]. In this context, natural organisms could provide possible solutions to the grand challenges in the development of 3D flexible devices and functional systems. Figure 1 offers an overview of the design and manufacture of 3D bioinspired flexible devices, involving three key aspects, i.e. exploration of bioinspired designs, development of manufacturing methods, and applications in practical scenarios. Specifically, it is crucial to elucidate microstructural constructions and structure-function principles of target natural organisms (e.g. animals, plants and microorganisms), which typically require multidisciplinary approaches (e.g. morphological observation, biological anatomy, microscopic characterization and mechanical testing). The development or selection of suitable manufacturing methods is fundamental for the realization of 3D flexible devices. In recent years, enormous progress can be seen in 3D fabrication methods of bioinspired devices, including advances in direct 3D manufacture (e.g. 3D printing, laser ablation and templating) and 2D-to-3D assembly (e.g. soft active materials-based actuation methods and mechanically guided 3D assembly) [2,28–31]. Additionally, 3D bioinspired flexible devices (e.g. sensors, energy harvesters, optoelectronics, soft robotics and biomedical devices) are used in target practical scenarios for environmental perception, drug delivery and health monitoring, among other applications. Note that bioinspired 3D flexible devices discussed in this paper typically refer to the flexible devices constructed with 3D bioinspired structures based on clear structure-function–coupling principles, where the feature sizes of functional units along three dimensions are comparable to each other. A few existing reviews have covered related topics on bioinspired design strategies, including bioinspired heterogeneous materials and actuators [23,29,32], while comprehensive reviews on bioinspired designs of 3D flexible devices and functional systems are still lacking.

In this review, we highlight the latest progress in bioinspired 3D flexible devices and functional systems, with a focus on the design concepts, manufacturing methods, structure-function principles and applications. It begins with an overview of the physical characteristics of soft natural organisms and a summary of 3D manufacture methodologies in the next section, followed by discussions on design strategies and applications of bioinspired 3D flexible sensors, energy harvesters, optoelectronics, soft robotics and biomedical devices. Conclusions and an outlook on the existing challenges and potential solutions are provided in the last section.

## OVERVIEW OF SOFT NATURAL ORGANISMS AND 3D MANUFACTURE METHODOLOGIES

In nature, the incomparable diversity of biological organisms provides an endless source of inspiration for the design of bioinspired 3D flexible devices and functional systems. However, it is challenging to impart biological functions into man-made systems, due to the remarkably wide dispersion of physical characteristics (e.g. feature sizes, topological complexities, mechanical properties and locomotion speeds) for natural organisms. 3D manufacture methodologies based on advanced processing techniques and functional materials provide a pivotal bridge between bio-inspirations and 3D flexible devices/systems. In this section, representative physical characteristics of selected natural organisms are quantitatively summarized, followed by an overview of typical 3D manufacturing methods, providing basic knowledge for the design, fabrication and application of 3D bioinspired devices/systems in different areas.

### Quantitative summary of physical characteristics of natural organisms

**Figure 2.**
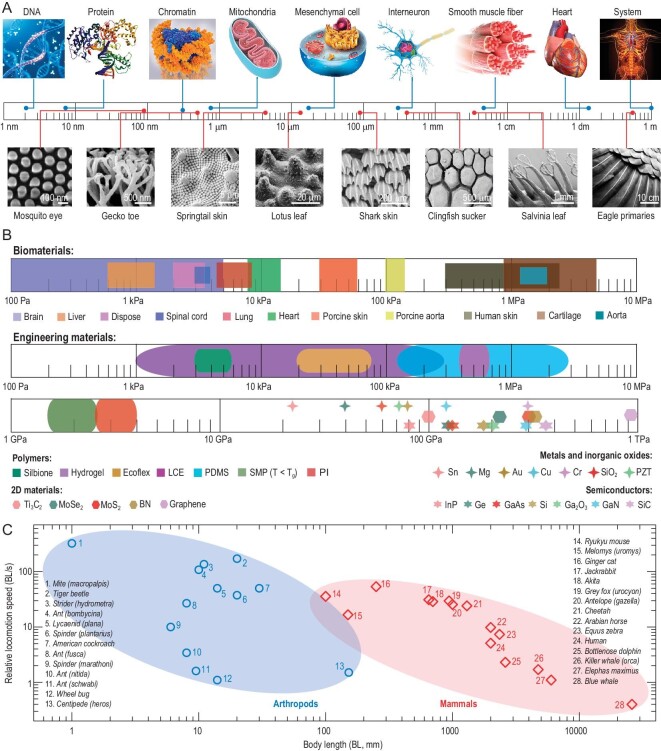
A quantitative summary of representative physical characteristics of natural organisms. (A) Feature sizes (top) and surface morphologies (bottom) of various biological structures. SEM images of surface morphologies are adapted with permission from Ref. [32]. Copyright 2009 Royal Society Publishing. (B) Young's moduli of various biological materials and typical engineering materials used in flexible electronic devices. (C) Plot of relative locomotion speeds of various mammals and arthropods versus their body lengths (BLs).

Figure 2 provides a quantitative summary of characteristic sizes (Fig. 2A, top), surface morphologies (Fig. 2A, bottom), Young's moduli (Fig. 2B) and locomotion speeds (Fig. 2C) of selected natural organisms that have been widely studied in the fields of bioinspired materials, flexible devices and biomedical engineering.

Feature sizes and hierarchical geometries of selected biological structures in the human body are plotted in the top panel of Fig. 2A, which shows a wide size dispersion from the nanometer- to meter-scale. For example, the diameter of a double helix in deoxyribonucleic acid (DNA), lateral size of the mesenchymal cell and longitudinal diameter of an adult human heart are ∼2 nm, ∼18 μm and ∼14 cm, respectively. Besides, 3D hierarchical geometries already exist widely in biological structures, and their complexity increases with increasing feature size, as evidenced by the multi-level construction of the human body, i.e. biological macromolecules-organelles-cells-tissues-organs-systems. Typically, the physiological functions of biological structures are closely related to their feature sizes and specific 3D shapes, which are two key variables to consider when designing bioinspired 3D flexible devices. The bottom panel of Fig. 2A presents some typical surface morphologies of biological organisms, such as the trichomes on the salvinia leaf, the placoid scales on the skin of a shark, the mastoids on the lotus leaf, the setae on the gecko toe and the nano-nipples on the mosquito eye [32]. These multi-scale, hierarchical surface morphologies are particularly crucial to hydrophobicity, hydrophilicity, reversible adhesion, drag reduction, energy conversion, light coloration, and many other biological functions, providing valuable inspiration for the design of 3D flexible sensors, optoelectronics and energy harvesters. By tuning the surface morphologies, some natural organisms exhibit very low Poisson ratios, such as the overlapping and interlocking scales of the Arapaima gigas fishes, pangolins and pinecones, which tend to open up under external loading. Such low Poisson ratios or even auxetic behaviors could be introduced in the design of 3D soft crawling robotics based on morphable scales and mechanical energy harvesters.

Figure 2B shows distributions of Young's moduli (*E*) of selected soft biological tissues and commonly used engineering materials (in flexible devices) within the wide ranges of [100 Pa, 10 MPa] and [100 Pa, 1 TPa], respectively [23]. Note that Young's moduli of soft tissues/materials (e.g. the skin and Ecoflex silicone) with evident nonlinear deformation characteristics are derived from the initial moduli in mechanical tests. Obviously, the key electronic materials (e.g. copper and silicon) widely used in 3D flexible electronics are much stiffer (e.g. modulus ratio >1000 times) than biological soft materials, and such a significant modulus mismatch is important to consider in the design of bioinspired devices and functional systems. Possible solutions lie in developing elaborated structural design strategies (e.g. kirigami, origami, fractal and 3D buckling transformation) based on traditional stiff materials, or exploring new synthesis methods for biomimetic soft materials which have conducting or semiconducting properties [2,33]. Besides, employing natural soft materials (e.g. the cocoon fiber, sponge, cotton and feather) with or without modifications in 3D flexible devices and systems holds several advantages over engineering materials, aligning with both environmental and functional considerations. The excellent biodegradability and sustainability of natural soft materials would be crucial in addressing the growing concern of electronic waste, and their mechanical flexibility and light weight would open up new possibilities for 3D flexible devices. Besides, the protein-based materials (e.g. collagen, gelatine and fibrin) and polysaccharide-based materials (e.g. cellulose, chitosan and alginate) are inherently biocompatible, and would be less likely to cause adverse reactions in contact with living cells and tissues, which is particularly important for biomedical devices.

An efficient locomotion, characterized by the locomotion speed (BL/second) relative to the body length (BL), is indispensable for animal survival. Depending on different living environments, a variety of distinct anatomical structures and locomotion modes have evolved in animals. For example, humans, bony fishes and birds rely on legs, fins and wings for terrestrial running, aquatic swimming and aerial flying, respectively. Figure 2C summarizes the relative locomotion speeds and corresponding body lengths (BLs), clearly showing two distinct distribution ranges for mammals and arthropods. Specifically, arthropods generally possess a smaller body length than mammals, while the relative speeds of many arthropods are much greater than that of mammals. For example, the California mite has the fastest relative speed (up to 322 BLs per second) among all animals, while the cheetah has a relative speed of only 16 BLs per second. The locomotion behaviors found in different animals could provide excellent examples to inspire new designs of 3D soft robots, less-invasive interventional operations and *in vivo* drug delivery.

Excluding the four features discussed above, other physical characteristics of natural organisms could also be very interesting to reproduce in artificial materials/systems. For example, 3D cellular structures with high porosities widely exist in many living organisms, contributing to their low-density and lightweight feature. Balsa wood consisting of a network of thin-walled cells is renowned for its exceptionally low density and high porosity, and sea sponges with a porous skeletal structure are capable of efficiently filtering and circulating water. The 3D cellular structures could provide valuable inspiration for novel designs of 3D flexible devices, such as the 3D lightweight microfliers and 3D flexible resistive sensors with a high sensitivity.

### Manufacture methodologies of bioinspired 3D flexible devices and systems

The physical characteristics derived from natural organisms as described above, including feature sizes with a wide spanning, structural topologies with a high complexity, surface morphologies with hierarchical patterns, soft materials with very low Young's moduli, and distinct deformation/locomotion modes, pose great challenges in the design and fabrication of bioinspired 3D flexible devices and systems. In recent years, enormous progress in 3D manufacturing methods have been made, with the rapid development of new techniques and the emergence of new functional materials. The manufacture methodologies of bioinspired 3D flexible devices/systems can be classified into two broad classes, including direct 3D manufacture and 2D-to-3D assembly. Representative methods, processing techniques, applicable materials, advantages, limitations and typical applications are summarized in Supplementary Table S1 in the online Supplementary file.

3D printing, laser ablation and templating are three representative direct 3D manufacturing methods, in which 3D biomimetic structures are formed in one processing step without involving any 2D intermediate precursors. Specifically, based on well-developed 3D printing techniques such as direct ink writing (DIW), fused deposition modelling (FDM), digital light processing (DLP), two-photon lithography (TPL), large-area projection micro-stereolithography (LAPμSL) and continuous liquid interface production (CLIP), 3D biomimetic structures with arbitrary geometries and wide feature sizes can be realized in a bottom-up process. More discussions on 3D printing can be found in a number of reviews [28,29], and are not elaborated in the present review. Although several recent works developed new 3D printing technologies for metal materials (e.g. liquid metal and silver gel), the most prominent challenge of applying 3D printing in 3D bioinspired flexible electronics still lies in accessible printing materials. At this stage, 3D printing of inorganic semiconductor materials (such as the monocrystalline silicon) is still elusive. Featuring efficient and high accuracy in material removal capability, laser ablation can be leveraged to form 3D biomimetic structures through cutting, engraving or surface tuning for almost all types of materials used in flexible electronics [34,35]. Fabrication of micro-lenses in an insect-inspired compound eye camera serves as an example of successful application [34]. Templating methods are capable of directly shaping 3D biomimetic structures by casting, coating or soft lithography based on predefined templates, with the length scale ranging from hundreds of micrometers to dozens of centimeters [36]. However, templating methods are incompatible with traditional electronic materials (e.g. metals and inorganic semiconductors) due to their ultrahigh Young's moduli.

The 2D-to-3D assembly represents an alternative path to manufacture 3D bioinspired flexible devices and functional systems, which involves the preparation of 2D precursor structures with pre-designed patterns, and the subsequent 3D assembly is driven by various types of forces [2]. Soft active materials can autonomously undergo out-of-plane deformations under specific stimuli, thus providing opportune actuation forces for 3D assembly. Based on different actuation stimuli and deformation mechanisms, a number of active actuation methods have been developed, including swelling, thermal, light and electromagnetic actuations, which are widely used in the assembly of biomimetic 3D structures [2,28,29]. Specifically, swellable materials (e.g. hydrogels and hydrogel-filler composites) can be patterned into 2D precursor structures, and then transformed into 3D configurations with a change of the ambient humidity. Featuring substantial thermal-induced deformability, phase-change materials, such as shape memory polymers/alloys (SMPs/SMAs) and liquid crystal elastomers (LCEs), are highly suitable for use in bioinspired soft actuators and robotics. Light-based actuation relies on soft polymers with light-absorbing nanoparticles (e.g. graphene and carbon nanotubes) or photoactive groups (e.g. azobenzene, anthracene, diarylethene and fulgide), which involves deformations induced by photochemical reactions. Electro-magnetically coupled actuation depends either on interactions between magnetic fields and soft polymers embedded with ferromagnetic particles, or on Lorentz forces applied on flexible interconnects. By embedding microchannels in elastomers, fast and reversible shape morphing can be realized based on pneumatic actuation, while such devices are typically tethered by air pipes. Note that 2D-to-3D assembly methods for bioinspired flexible structures/devices based on soft active materials and pneumatic actuations typically suffer from the incompatibility issue with electronic materials. By exploiting buckling deformations of 2D precursor structures driven by in-plane compression or tension, the recently developed mechanically guided 3D assembly provides a solution to this issue [1,2]. Appealing features of this scheme include the broad applicability to nearly any type of material (e.g. inorganic semiconductors, metals, polymers and various heterogeneous combinations), over length scales from sub-micrometer to meter dimensions, the rich diversity of accessible 3D geometries, and the excellent compatibility with planar microfabrication techniques.

## BIOINSPIRED 3D FLEXIBLE SENSORS

Instant and sensitive perception of surrounding environments is one of the essential abilities for varieties of natural organisms. By introducing unique biological structures (e.g. twining microfibers, curvy microcracks, hierarchical micropatterns and cellular microstructures) and structure-function principles into the design of 3D flexible sensors, outstanding performances, such as highly sensitive detection, multichannel sensing and large-area measurements, could be realized. Depending on specific application scenarios, the 3D flexible sensors could adopt different mechanisms for pressure/strain measurements, including the piezoresistive, piezoelectric, ferroelectric, electromagnetic, capacitive and triboelectric effects [8,9,37–39]. In recent years, remarkable progress has been made in the design and fabrication of bioinspired 3D flexible sensors, with practical implications for the Internet of Things (IoT), haptics and health monitoring. A number of representative 3D bioinspired flexible sensors and their sensing mechanisms, bionic structures, materials, sensitivity, measuring range and robustness are summarized in Table 1.

**Table 1.** Performance metrics of representative 3D bioinspired flexible mechanical sensors.

**Categories**	**Mechanisms**	**Bionic 3D structures**	**Materials**	**Sensitivity**	**Measuring range**	**Robustness**
**Twining micro-fibers**	Piezoresistive	Cytoskeleton-like nanofibers [22]	Si nanowire	∼9.6 × 10^−5^ kPa^−1^ Gauge factor: ∼760	Pressure: 0 to ∼40 kPa Strain: 0 to ∼0.01%	>10 000 cycles
	Capacitive	Double helical fibers [8]	AgNPs embedded in polyurethane fibers	Gauge factor: ∼12	Strain: 0 to ∼27.5%	>2000 cycles (at 10% strain)
**Curvy micro-cracks**	Piezoresistive	Scorpion–slit-like curvy crack array [15]	AgNPs coating on PDMS	Gauge factor: ∼1800	Strain: 0 to ∼0.65%	>7000 cycles (at 0.2% strain)
	Piezoresistive	Stereocilia bundle-like cracks [40]	Pt coating on PDMS nanowires	Gauge factor: ∼2.6 × 10^5^	Strain: 0 to ∼130%	>1500 cycles (at 50% strain)
	Capacitive	Origami-based 3D microcracks [41]	Parylene/Au/Parylene on SU8 panels	Gauge factor: ∼0.44	Strain: 0 to ∼200%	>700 cycles (at 100% strain)
**Hierarchical micro-patterns**	Piezoresistive	Corpuscle-like multilayer patterns [42]	Carbon black embedded in porous TPU film	Resolution: ∼1.8° for polar angle	Angle: 0 to ∼90°	>15 000 cycles (at 0.4 Hz)
	Piezoresistive	Interlocking beetle-inspired patterns [9]	Pt coating on polymer nanohairs	Gauge factor: ∼11.45	Pressure: 0 to ∼1.5 kPa	>10 000 cycles (at 1.5 kPa)
	Piezoelectric	Finger-like multilayer patterns [44]	PDMS pillars on PVDF film	∼346.5 pC·N^−1^	Force: 9 to ∼4300 mN	>2.16 × 10^6^ cycles (at 4.3 N)
	Capacitive	Skin-like hill array and pyramid array [38]	Carbon nanotubes coating on polyurethane film	∼0.19 kPa^−1^ for pressure <1 kPa	Pressure: 0 to ∼100 kPa	>30 000 cycles (at 15 kPa)
	Triboelectric	Leaf cone patterns with tinny burrs [37]	AgNWs coating on polymer film	127.22 mV·kPa^−1^	Pressure: 5 to ∼50 kPa	>5000 cycles (at 25 kPa)
**Cellular micro-structures**	Piezoresistive	Urchin-like 3D microspikes [45]	ZnO particles sandwiched between ITO/PET films	∼121 kPa^−1^ Gauge factor: >10^4^	Pressure: 0 to ∼10 kPa Strain: 0 to ∼0.8%	>3000 cycles (at 150 Pa)
	Piezoelectric	Multilayer fur-like cage cellular structures [39]	PVDF and Au film on 3D polyimide frames	∼1.8 V·N^−1^ for force <0.025 N	Force: 0 to ∼0.1 N	>1000 cycles (at 32% strain)
	Capacitive	Lotus leaf mastoid-like cellular structures [47]	Ultraslippery coating on 3D gold electrodes	79.1 ± 4.3 pF·kPa^−1^	Pressure: 0 to ∼15 kPa	>1000 cycles (at 5 kPa)
	Capacitive	Flea leg-like 3D cellular structures [46]	Silver coating on PDMS films	∼1.005 kPa^−1^ for pressure <1 kPa	Pressure: 0 to ∼200 kPa	>6000 cycles (at 10 kPa)

**Figure 3.**
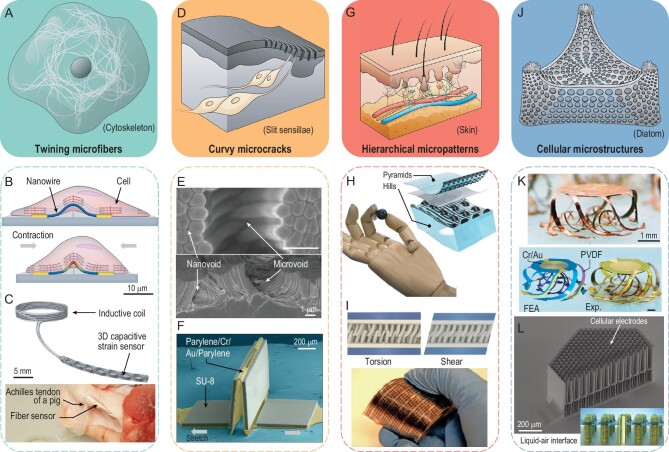
Bio-inspired 3D flexible sensors. (A) Illustration of twining microfibers (e.g. cytoskeleton) in a cell. (B) Illustration of a nanowire-based biosensor featuring simultaneous measurements of cell contraction force and cytomembrane action potentials. (C) A 3D capacitive sensor with double helical conductive fibers capable of wireless monitoring of tendon tissue strains. (D) Illustration of curvy microcracks on a scorpion leg. (E) SEM images of a strain sensor with stereocilia bundle-like Pt nanoclusters and micro-/nano-voids induced by the capillary force. (F) SEM image of a highly stretchable strain sensor with an origami-induced 3D microcrack. (G) Illustration of hierarchical micropatterns in human skin. (H) A prosthetic hand integrated with a hierarchically patterned electronic skin and an inset illustration of its 3D device structure. (I) Deformation modes (top) and optical image of a pressure sensor with 3D interlocking hierarchical microstructures inspired by the *Promethis valgipes* beetles. (J) Illustration of cellular microstructures in a diatom. (K) A 3D cage-shaped cellular mesostructure (top) and a 3D force/temperature sensor (bottom) inspired by the *Enhydra lutris* fur. (L) SEM image of a 3D pressure sensor with a lotus leaf mastoid-inspired pillar array structure and the ultra-slippery electrode surface. Adapted with permission from: (B) Ref. [22]. Copyright 2022 AAAS. (C) Ref. [8]. Copyright 2021 Springer Nature. (E) Ref. [40]. Copyright 2019 Springer Nature. (F) Ref. [41]. Copyright 2023 AAAS. (H) Ref. [38]. Copyright 2018 AAAS. (I) Ref. [9]. Copyright 2012 Springer Nature. (K) Ref. [39]. Copyright 2021 IOP Publishing. (L) Ref. [47]. Copyright 2023 Springer Nature.

Microfibers widely exist in natural organisms, such as the vascular fibers in plants, skeletal/smooth/cardiac muscle fibers in the human body and the cytoskeleton in cells (Fig. 3A), which have inspired the design of 3D flexible sensors with twining microfibers. By mimicking the biofibers in the extracellular matrix, a 3D nanowire-based nano-transistor was developed (Fig. 3B), which is capable of simultaneously measuring electrical and mechanical cellular responses based on the field effect and piezoresistive effect, respectively. Sub-millisecond, scalable tracking and differentiation of cardiac cell states after drug release were demonstrated based on the transistor sensor array [22]. The slender feature of the microfiber-based sensor facilitates implantable integrations with muscles, vessels and tendons. Figure 3C shows a suturable strain sensor consisting of capacitive double-helical fibers and an inductive coil [8]. The tensile deformations of achilles tendon and knee ligament in an *ex vivo* and *in vivo* porcine leg were measured and recorded based on wireless transmission.

Curvy microcrack mimicking the insect slit sensillum represents another bioinspired design of 3D flexible sensors (Fig. 3D). For example, inspired by the slit sensillum in scorpions, an omnidirectional and ultrasensitive strain sensor with circumferentially arrayed microgrooves was realized to monitor the low-frequency vibrations of bridges [15]. Utilizing the collapse and agglomeration of nanowires induced by capillary forces, cluster microstructures with micro-/nano-voids can be obtained, and microcracks initiated from these voids enable an ultrahigh stretchability (∼130%) and a high sensing resolution (∼0.005%) of tensile strains (Fig. 3E) [40]. By introducing the origami design, a controllable microcrack can be formed between two electronic panels based on the mechanically guided assembly process (Fig. 3F). The origami strain sensor exhibits ∼200% stretchability and ∼1.2% hysteresis, which is capable of accurately measuring large and dynamic deformations of a soft multimodal robotic arm [41].

Bioinspired hierarchical micropatterns are widely adopted in 3D flexible sensors, which find innovative design principles of structure-function relation from surface morphologies of plants and animals. For example, the human skin can measure pressure, vibration, temperature and humidity based on its hierarchical structures and different receptors (Fig. 3G), which has inspired the development of artificial electronic skin (e-skin). Mimicking spinosum microstructures of the dermis-epidermis interface, an e-skin hierarchically patterned with pyramids and hemispherical hills was fabricated, featuring decoupled measurements of normal and shear forces based on capacitive effects (Fig. 3H) [38]. By hierarchically arranging two piezoresistive sensing layers, an e-skin capable of measuring both the pressure and loading angles was realized, featuring a high measurement resolution of the polar angle (∼1.8°) and azimuthal angle (∼3.5°) [42]. Mimicking the biological path from skin mechanoreceptors to nerves, an artificial mechanoreceptor system was constructed based on a pyramid-patterned piezoresistive sensor, an organic transistor circuit and a photoelectric converter, exhibiting a sublinear response to increasing force stimuli [43]. Inspired by the hierarchical skeleton-tissue structure in the finger, a piezoelectric tactile sensor with rigid pillars and a flexible polyvinylidene fluoride (PVDF) film was fabricated, and the 3D hard-soft construction enables enhanced sensitivity at high frequency based on the d_31_ working mode in the PVDF film [44]. Hierarchical micropatterns can also be formed by the interlocking microstructures existing in lasso peptides, ginkgo husks, cochlear hair cells and beetle elytra. Figure 3I shows a piezoresistive sensor with interlocking nanofibers inspired by the elytra of beetles, capable of detecting ultralow pressure (∼5 Pa), shear force (∼0.001 N), and torsion force (0.0002 N·m) [9]. Similarly, based on the interlocking of sea urchin-like ZnO microparticles, a piezoresistive sensor exhibits a high sensitivity (121 μA kPa^−1^) and good robustness (>2000 cycles) [45].

3D cellular microstructures (e.g. the cellular shell of diatoms) are the fourth kind of bioinspired structural designs for flexible sensors (Fig. 3J), which could offer unusual capabilities (e.g. multistage sensing and underwater sensing) with optimized 3D geometries and material integrations. For example, Fig. 3K shows a 3D multifunctional cage-shaped sensor inspired by the multilayer fur of *Enhydra lutris*, which was fabricated based on the mechanically guided 3D assembly [39]. Relying on the integrated Au electrodes and PVDF film, this sensor is capable of simultaneously measuring ambient temperature and compressive forces, while its distinct bilayer cage-shape geometry enables multistage load-bearing and collapse prevention under extreme out-of-plane compressions. Similarly, mimicking the flea leg, a capacitive pressure sensor consisting of an array of 3D cellular arcs was realized, featuring a two-stage mechanical response [46]. Note that the measuring range and sensitivity of the two 3D flexible sensors with cellular structures can be well tailored by changing the ribbon width, thickness and radius of curved ribbons. Different from solid sensing modules in conventional pressure sensors, Fig. 3L shows a pressure sensor utilizing a trapped air layer to modulate capacitance changes, which is inspired by the air entrapment phenomenon of submerged lotus leaves [47]. The 3D cellular microstructures with an ultraslippery electrode surface enable an outstanding linear pressure sensing capability with ultralow hysteresis (∼1.34%) and high sensitivity (∼79.1 pF kPa^−1^).

## BIOINSPIRED 3D ENERGY HARVESTERS

In natural systems, many plants exhibit phototropism and are capable of converting light energy into bioenergy through the chlorophyll in an efficient manner. For instance, diatoms can enhance the efficiency of photosynthesis by trapping sunlight with 3D cellular architectures made of SiO_2_. In recent years, inspired by the energy harvesting behaviors of plants, animals and microorganisms, a number of artificial energy harvesters have been developed to convert light, thermal and mechanical energy to electrical energy, showing great potential in supplying energy for the low-power sensor systems in the fields of IoT and biomedical devices (especially implantable devices).

**Figure 4.**
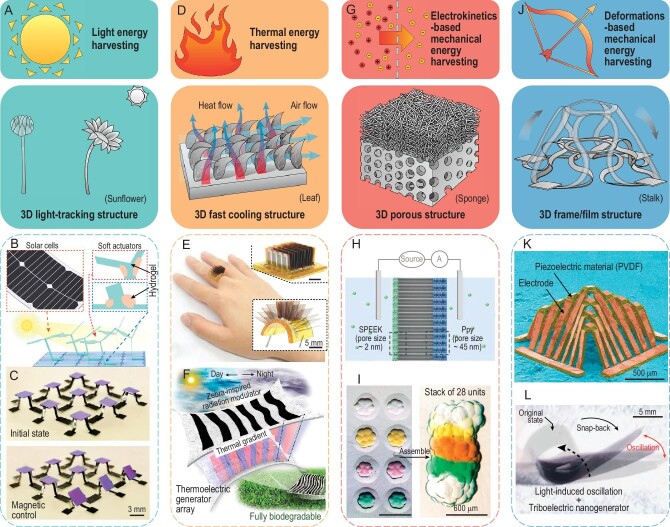
Bioinspired 3D energy harvesters. (A) Illustrations of light energy harvesting (top) and a 3D light tracking structure (bottom). (B) A bioinspired heliotropic structure actuated by the transpiration of the hydrogel-paper muscle, capable of harvesting solar energy by integrating solar cells [49]. (C) An array of magnetically responsive 3D mesostructures integrated with organic solar cells. (D) Illustrations of thermal energy harvesting (top) and a 3D fast cooling structure (bottom). (E) An array of leaf-inspired thermoelectric generators integrated on a finger, featuring self-healing and full recycling properties. (F) Illustrations of a biodegradable, stretchable thermoelectric system with a zebra-inspired radiation modulator. (G) Illustrations of mechanical energy harvesting based on electrokinetics (top) and a 3D porous structure (bottom). (H) Illustration of a salinity self-adaptive nanofluidic diode with a sandwiched porous membrane inspired by the euryhaline fish. (I) An electric-eel–inspired soft electrokinetic energy harvester by stacking 28 droplet-assembled power units. (J) Illustrations of mechanical energy harvesting based on 3D deformable structures (top) and a 3D frame structure (bottom). (K) SEM image of a 3D piezoelectric energy harvester. (L) optical images of a 3D triboelectric nanogenerator. Adapted with permission from: (C) Ref. [35]. Copyright 2021 Wiley-VCH GmbH. (E) Ref. [51]. Copyright 2021 AAAS. (F) Ref. [54]. Copyright 2023 AAAS. (H) Ref. [55]. Copyright 2022 Wiley-VCH GmbH. (I) Ref. [6]. Copyright 2023 Springer Nature. (K) Ref. [56]. Copyright 2019 Springer Nature. (L) Ref. [58]. Copyright 2020 Wiley-VCH GmbH.

The phototropism allows plants (e.g. the *Helianthus annuus*) to orient their stems/leaves/flowers toward the sun throughout the daytime, which can maximize the efficiency of light energy harvesting. Similarly, the 3D light-tracking structure plays an important role in the design and fabrication of 3D flexible energy harvesters for light energy (Fig. 4A). For example, an energy harvester based on nanostructured stimuli-responsive polymers is capable of automatically tracking the direction of light omnidirectionally and thus enabling up to a 400% solar energy-harvesting enhancement at oblique illumination angles [48]. By introducing hydrogel joints in an artificial plant, programmed stem tilting and leaf opening can be realized based on a light-induced deswelling process (Fig. 4B) [49]. This self-regulating artificial plant can be used to harvest solar energy in a controlled manner by integrating solar panels onto its leaves. Different from the relatively low actuation speed of hydrogel materials, magnetic actuation enables fast and reversible light-tracking behaviors of 3D solar energy harvesters with a 3D table-like geometry (Fig. 4C) [35]. Note that a large array of 3D energy harvesters integrating organic solar cells could be assembled parallelly based on the mechanically guided assembly method, showing the potential to achieve large area light-tracking–based energy harvesting.

Temperature differences exist nearly everywhere in both natural and man-made environments, while it remains challenging to create high temperature gradients for biointegrated flexible thermal energy harvesters. By introducing bioinspired 3D fast cooling structures and making full use of the heat exchange in body surface microenvironments, high efficiency of thermal energy harvesting could be realized (Fig. 4D). For example, a 3D flexible thermal energy harvester inspired by grass leaves is composed of an array (10 × 10) of vertically aligned cooling fins, which is capable of generating 11 μW when integrated on an arm at room temperature [50]. Based on a similar inspiration, another wearable thermal energy harvester with an array of modular thermoelectric chips is shown in Fig. 4E, featuring an ultrahigh open-circuit voltage (1 V/cm^2^) at a temperature difference of 95 K. The dynamic covalent thermoset polyamine in the substrate and encapsulation of the device contributes to full recycling after solution processing and self-healing following mechanical damage [51]. Based on the mechanically guided assembly method, an array of 3D thermal energy harvesters was fabricated, where p-type and n-type silicon ribbons integrate into compliant 3D helical architectures [52]. An increased efficiency of energy harvesting was demonstrated experimentally due to its efficient thermal impedance matching and multiplication of heat flow.

As climate change and global warming intensify, bioinspired radiative cooling technologies are attracting more attention due to their capabilities of reducing energy consumption in a passive and renewable manner. The 3D hierarchically photonic architectures of the hairs of Saharan silver ants can enhance reflectivity in both the visible and near-infrared spectrum ranges, and the emissivity in the mid-infrared range, thus allowing their efficient thermoregulation and survival in the hottest and driest environments. Introduction of the bioinspired passively radiative cooling to the flexible devices enables novel applications in energy harvesters and personal thermal management [53]. For example, by integrating a zebra stripe-shaped, stretchable radiative modulator with an array of n-/p-doped Si nanomembranes, all-day sustainable thermal energy harvesting with an unexpected level of in-plane temperature gradient was realized (Fig. 4F). Note that this flexible system is capable of working under external mechanical stretching, and is fully biodegradable under physiological conditions [54].

Electrokinetic energy harvesting systems based on 3D nanopores and microchannels provide a new path to convert fluidic mechanical energy to electrical energy (Fig. 4G). For example, inspired by the euryhaline fishes, a high-performance salinity-gradient power generator was fabricated (Fig. 4H), which integrates two composites [sulfonated poly(ether ether ketone) (SPEEK) and polypyrrole (Ppy)] into two sides of an anodic aluminum oxide (AAO) template. The porous morphology of one side of the AAO can adapt to changing salinity and switch from straight nanochannels (pore size ∼45 nm) to 3D networks (pore size ∼2 nm), thus inducing three orders of magnitude change for charge density [55]. By introducing bioinspired microstructures for light-trapping and porous ionic hydrogel for water supply, an energy harvester based on the electrokinetic effect was demonstrated, which can generate electricity using the evaporation of water [7]. Recently, a miniaturized soft power source inspired by the electric eel was fabricated with lipid-supported 3D porous networks of hydrogel droplets, which can generate electricity based on internal ion gradients (Fig. 4I) [6]. Modulation of the neuronal network activity in 3D microtissues was demonstrated based on this bioinspired energy device.

Mechanical energy harvesting based on the deformations or dynamic movements of bioinspired 3D frame/film structures represents a widely investigated area, which could involve different mechanisms (e.g. the piezoelectric effect, triboelectric effect and electromagnetic effect) according to their application scenarios (Fig. 4J). 3D piezoelectric microstructures made of PVDF materials can be realized based on the mechanically guided assembly process (Fig. 4K), and their complex 3D topologies enable more conversion modes for mechanical energy harvesting in the human body compared with their 2D counterparts [56]. Triboelectricity has attracted wide interest in energy harvesting due to its structural simplicity and wide applicability. For example, a jellyfish-inspired triboelectric nanogenerator was used to harvest sea wave energy with an output performance of 143 V, 11.8 mA/m^2^ and 22.1 μC/m^2^ under a low frequency of 0.75 Hz vibration [57]. Figure 4L shows an autonomous droplet-shaped energy harvester based on the triboelectric effect, which undergoes light-driven oscillating motions [58]. Specifically, in one deformation cycle, the thin-film device transforms from a planar configuration to a rolled-up configuration under photomechanical actuation, then snaps back due to the reduction of illumination area, and finally oscillates around an equilibrium position to generate electrical energy.

## BIOINSPIRED 3D OPTOELECTRONICS

For better locomotion and predation, various animals have evolved extraordinary photonic structures, such as the antireflecting moth eyes, motion-sensitive dragonfly compound eyes, self-luminous firefly lanterns, iridescent tiger beetles and butterfly wings. Such photonic structures and functions have been inspiring the development of artificial 3D optoelectronics for advanced light imaging, light extraction and structural coloration. Bioinspired 3D electronic eyes with single-chambered or compound layouts represent a source of increasing interest from diverse areas, and play crucial roles in the booming intelligent perceptions for soft robots, human prostheses and self-driving vehicles.

High-resolution eyes with a 3D single-chambered layout widely exist in vertebrates and chelicerates, which are typically composed of a single lens, a curvy retina and light-adaptation tissues/organs (e.g. the cornea, iris and lens in human eyes). Remarkable imaging capabilities of single-chambered eyes [e.g. 95° field of view (FoV), tunable focal length and high resolution (∼100 μm) for human eyes] inspired the development of novel artificial eyes. To mimic the undevelopable retina structure and decrease the pixel size, a variety of design strategies have been devised for conformal integration of photodetectors or image sensors with 3D curvilinear substrates/tissues, including the curvy mesh, kirigami/origami and nanoarray designs.

**Figure 5.**
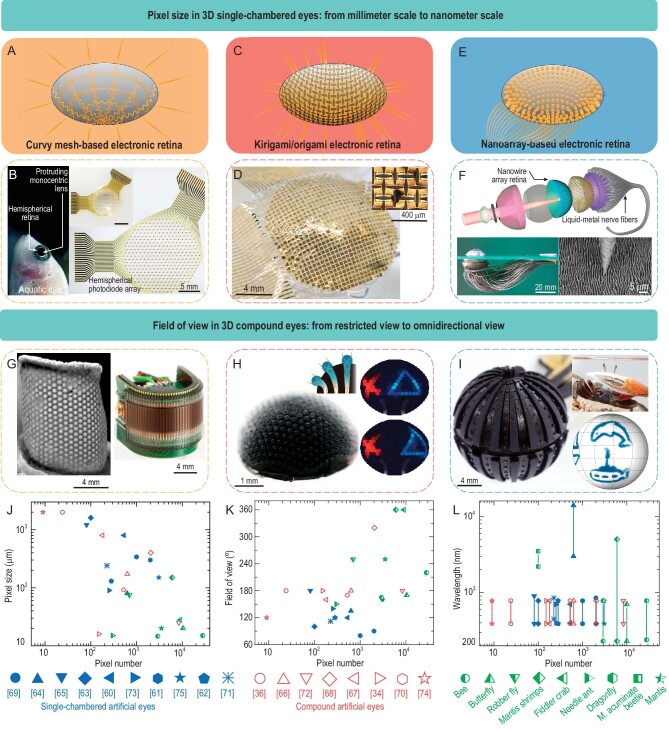
Bioinspired 3D electronic eyes. (A) Illustration of a curvy mesh-based electronic retina. (B) An aquatic-vision–inspired camera with a monocentric lens and a silicon nanorod photodiode array. (C) Illustration of a kirigami-based electronic retina. (D) A convex camera with kirigami pixel designs. (E) Illustration of a nanoarray-based electronic retina. (F) Exploded view (top) and optical image (bottom left) of an electronic eye with a hemispherical perovskite nanowire array retina (bottom right). (G) The compound eye of the extinct *trilobite* (left) and a curved artificial compound eye camera (right). (H) An apposition compound eye camera mimicking the eye of Asian needle ants (left) and reconstructed images showing the movement of a triangle (right). (I) An amphibious artificial compound eye camera inspired by the fiddler crab eye (left), featuring a panoramic visual field (right). (J– L) Pixel sizes (J), field of views (FoVs) (K) and wavelengths (L) of natural and artificial eyes versus pixel numbers. Adapted with permission from: (B) Ref. [60]. Copyright 2020 Springer Nature. (D) Ref. [61]. Copyright 2021 Springer Nature. (F) Ref. [63]. Copyright 2020 Springer Nature. (G) Ref. [66]. Copyright 2013 National Academy of Sciences. (H) Ref. [36]. Copyright 2021 Springer Nature. (I) Ref. [68]. Copyright 2022 Springer Nature.

The curvy mesh layout is the first kind of design strategy for 3D bioinspired electronic retinas, which involves a 3D interconnected network connecting rigid photodetectors on curved elastomeric substrates (Fig. 5A). For example, mimicking the single-chambered layout of human eyes, a tunable hemispherical electronic eye camera was realized, featuring reversible deformations of the photodetector array and fluidic planoconvex lens adjusted dynamically via hydraulics [59]. A 3.5× adjustable zoom capability of this eye camera was experimentally verified by coordinatively controlling the liquid pressure in the lens and photodetector array. Compared to the limited FoV of human eyes, the aquatic-type vision widely found in fish and aquatic mammals is capable of affording a wide FoV up to 160°. Figure 5B shows an aquatic-eye–inspired camera consisting of a single monocentric lens and a hemispherical artificial retina made of a silicon nanorod photodiode array [60]. The core-shell lens in this device mimics the parabolic reflex index profile in the protruding lens of an aquatic eye, which enables 120°-wide FoV imaging. Note that the sensor density and image resolution are significantly reduced due to the fact that the buckled interconnect network occupies part of the photosensitive area.

To increase the image resolution and decrease the pixel size, advanced design strategies of the artificial retina based on kirigami/origami (Fig. 5C and D) and nanoarray (Fig. 5E and F) have been developed. For example, a shape-adaptive camera with high pixel fill factors (∼78%) was fabricated by transferring a 32 × 32-pixel array of silicon optoelectronic pixels with a kirigami design onto curvy surfaces using conformal additive stamp printing (Fig. 5D) [61]. Alternatively, a bioinspired hemispherical camera with 281 photodetectors in a dense array can be fabricated based on the origami deformations of polygon blocks [62]. Figure 5F presents an electrochemical eye camera with a hemispherical retina made of high-density array of perovskite nanowires (similar to the photoreceptors, i.e. rod and cone cells of the human retina) [63]. By exploiting the nanowire-based strategy, a single pixel with 500 nm lateral size and a footprint of ∼0.22 μm^2^ was realized.

High-resolution biological vision in the invisible spectrum [e.g. infrared radiation (IR)] represents an unusual capability found in a few species (e.g. snakes and bullfrogs) in nature. A pit organ–inspired, hemispherical infrared camera with 625 pixels based on high-density ionic thermoelectric polymer nanowire arrays was demonstrated, featuring an ultrawide FoV up to 135° for mid- to long-infrared imaging of different temperature objects in a self-powered manner [64]. By spray-coating quasi-2D phenylethylammonium/formamidinium lead halide (PEA_2_FA_n-1_Pb_n_X_3n+1_) perovskite onto a hemispherical substrate, a bioinspired photodetector was realized with wavelength selective responses ranging from the visible to near-infrared range [65].

Biological eyes with a compound layout, widely existing in arthropods, are typically composed of an array of ommatidia on a convex hemisphere, with capabilities of providing extremely wide FoVs, high motion sensitivity, negligible geometrical distortion/aberrations and nearly infinite depth of field. Due to independent imaging of individual ommatidium, biological compound eyes afford an ideally dynamic vision, and therefore are very suitable for fast motion perceptions. For example, by mimicking the compound microstructures of the extinct trilobite Erbenochile erbeni, an artificial camera was fabricated, featuring a FoV of 180° × 60°, high temporal resolution and local adaptation to illumination (Fig. 5G). Precise measurements of angular velocities ranging from 50° to 358° per second at different ambient light levels were demonstrated [66]. By transfer printing a stretchable array of photodiodes and blocking diodes onto a prestretched substrate with a micro-lens array, a compound eye camera was assembled after releasing the prestrain [67]. This eye camera has a comparable number (180) of ommatidia comparable to those of Fire ants’ eyes and Bark beetles’ eyes. Alternatively, an apposition artificial camera mimicking the Asian needle ant eye is shown in Fig. 5H, in which 522 micro-lenses were strategically patterned across a hemispherical surface based on 3D printing with microfluidic-assisted molding [36]. The advantages of wide visible angle (up to 170°) and motion-based sensing of this camera were experimentally demonstrated.

Biological eyes in terrestrial and aquatic environments have inspired the development of various artificial visual systems, while amphibious vision systems received much less attention. Figure 5I shows an amphibious eye camera mimicking the compound eyes of a fiddler crab, where a flat surface is adopted in micro-lenses to maintain the same focal length whether in water or in air [68]. The comb-shaped silicon photodiode arrays were integrated into a spherical structure, thus affording an extremely wide FoV (up to ∼320°).

Fig. 5J–L summarizes key parameters of a number of representative 3D bioinspired eye cameras [34,62,64,65,67,69–75], including the pixel size, pixel number, FoV and wavelength for the perception spectrum. Pixel sizes of single-chambered artificial eyes are typically larger than 80 μm, while some artificial compound eyes can realize similar pixel sizes with the eyes of biological arthropods. The FoVs of artificial compound eyes are typically much larger than those of single-chambered artificial eyes. Therefore, future research efforts can concentrate on pursuing smaller pixel size (<30 μm), more pixel number (>10^3^) and larger FoVs (∼180° and ∼360° for single-chambered and compound artificial eyes, respectively). Besides, there is still very limited work on high-resolution 3D vision systems in the invisible light spectrum, implying rich research opportunities.

## BIOINSPIRED 3D SOFT ROBOTICS

Natural organisms living in different environments exhibit diverse locomotion modes, such as crawling, rolling and jumping in the terrestrial environment; swimming, floating and cilia-based swing in the aquatic environment; and flying, hovering and passive falling in the aerial environment. Inspired by such environmentally adaptive locomotion modes, a variety of 3D soft robotics have been developed based on advanced actuation strategies, including pneumatic/hydraulic pressures, soft active materials (e.g. SMPs, SMAs, LCEs and hydrogels), chemical reactions, and electromagnetic interaction-based actuations. These bioinspired soft robotics, categorized into 3D terrestrial/aquatic/aerial and amphibious robotics, present potential capabilities of implementing complex tasks encompassing inspection, search and rescue in confined spaces, covert surveillance, *in vivo* drug delivery and intelligent human assistance.

### 3D terrestrial soft robotics

**Figure 6.**
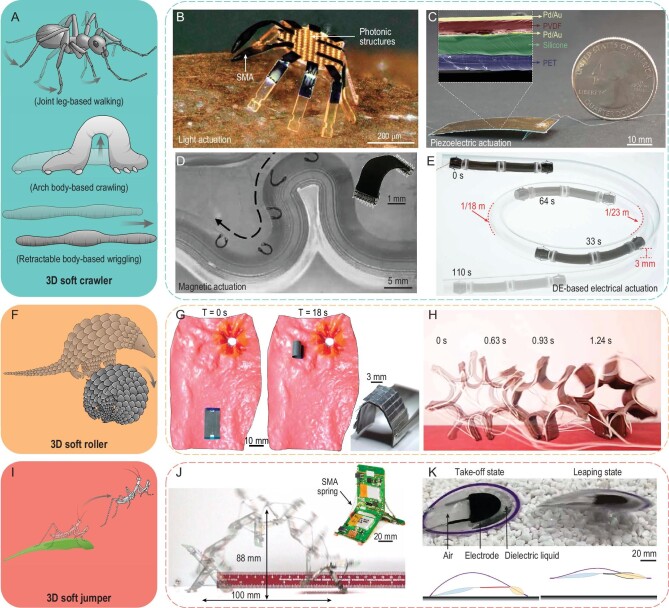
Bioinspired 3D soft terrestrial robotics. (A) Illustrations of three representative locomotion modes of 3D soft crawlers: leg-based walking (e.g. ants, top), arch body-based crawling (e.g. caterpillars, middle) and retractable body-based wriggling (e.g. earthworms, bottom). (B) A submillimeter-scale robot mimicking the peekytoe crab capable of laser-guided directional locomotion. (C) An insect-scale robot with the piezoelectric membrane body mimicking cockroaches. (D) A magnetically actuated climbing robot with bioinspired footpads capable of traversing confined spaces. (E) A pipeline inspection robot featuring navigating capability in sub-centimeter tubular environments. (F) Illustration of the pangolin-like rolling locomotion of 3D soft rollers. (G) An untethered magnetic robot capable of rolling in the stomach and on-demand biomedical heating. (H) A soft rolling robot actuated by shape memory alloy (SMA) wires. (I) Illustration of the mantis-like jumping locomotion of 3D soft jumpers. (J) A trap-jaw-ant–inspired jumping robot based on the snap-through mechanism. (K) A legless soft robot based on dielectric actuators capable of rapid and steered jumping. Adapted with permission from: (B) Ref. [77]. Copyright 2022 AAAS. (C) Ref. [79]. Copyright 2019 AAAS. (D) Ref. [80]. Copyright 2022 AAAS. (E) Ref. [82]. Copyright 2022 AAAS. (G) Ref. [19]. Copyright 2023 Springer Nature. (H) Ref. [83]. Copyright 2018 AAAS. (J) Ref. [84]. Copyright 2019 Springer Nature. (K) Ref. [85]. Copyright 2021 Springer Nature.

Crawling represents an important and widely adopted locomotion behavior for animals, which can be categorized into three representative modes, including leg-based walking (e.g. ants and the human), arch body-based crawling (e.g. caterpillars and hydras) and retractable body-based wriggling (e.g. earthworms and snakes) (Fig. 6A).

Crawling locomotion of 3D terrestrial soft robots in the micrometer scale is greatly constrained due to the strong adhesion induced by van der Waals and capillary forces [21], thus demanding the exploration of novel device design strategies and actuation mechanisms. For example, by adopting LCE membranes as artificial micro-muscles, a light-fueled microcrawler was fabricated [76]. Based on ∼20% contraction of the LCE muscle after laser beam heating, the crawler performed the wriggling locomotion on different surfaces (e.g. the polyimide coated surface and the grating surface). Alternatively, by utilizing SMA as the actuation material, submillimeter crawlers with complex 3D geometries (e.g. arrays of filaments, origami constructs and the crab-like configuration) were realized through the mechanically guided assembly process (Fig. 6B) [77]. The crab-like robot shows diverse locomotion modes, including walking and turning controlled by remote laser heating.

For bioinspired 3D millimeter-scale soft robots, limitations on device design and actuation methods imposed by body lengths are markedly reduced, thereby bringing out more unique 3D configurations and application scenarios. A soft tethered multigait crawler, consisting of electro-adhesive footpads, stiffness-variable smart joints and a deformable body, was demonstrated, which is capable of climbing on flat/curved surfaces and transitioning between two surfaces [78]. An insect-scale, piezoelectrically actuated robot inspired by the cockroach is shown in Fig. 6C, featuring a relative speed of 20 BLs/second, and excellent robustness to withstand 1 million times heavier loads than that of the robot [79]. Magnetic actuation of 3D bioinspired untethered robots offers safe and effective manipulation for biomedical applications within confined spaces. Figure 6D shows a soft robot consisting of a programmed ferromagnetic domain and adhesive footpads with hookworm-inspired microspikes, which can vertically climb 3D tissue surfaces covered by mucus and retain on unstructured 3D biosurfaces for a long period of time [80]. Cylindrical bodies of the annelid (e.g. earthworms and leeches) are typically composed of multiple self-similar soft segments and carry seta or parapodia to assist wriggling locomotion, which provides a wealth of natural design concepts for soft tubular robots. For example, a magnetically actuated origami crawler was realized, featuring body contraction induced by Kresling origami deformations [81]. By introducing dielectric elastomer actuators as artificial muscles and anchoring units as transmissions, a pipeline inspection robot was fabricated with a weight of 2.2 g and a length of 47 mm (Fig. 6E), featuring rapid crawling with ∼1.19 BLs/second horizontal speed and ∼1.08 BLs/second vertical speed [82].

Rolling-based locomotion usually endows animals with a higher moving speed, and an interesting example is the rolling of a pangolin in escaping predators (Fig. 6F). A pangolin-inspired bi-layered soft robot was fabricated (Fig. 6G), and it can roll in the intestinal tract with magnetic actuation and perform localized Joule heating with a temperature increase of >70°C within a short period of time (<30 seconds) [19]. Initial biomedical practices based on this untethered roller were demonstrated, including the mitigation of bleeding, hyperthermia and selective drug release. By embedding U-shaped SMA wires between prestretched and nonstretched layers of a thermally conductive elastomer, a lightweight (∼3 g) bionic roller is capable of generating rapid motions (maintaining a maximum speed of ∼8 mm/s for over 25 min) and forces (carrying more than 30 g of payload) comparable to natural muscles (Fig. 6H) [83].

In addition to the crawling and rolling locomotion modes discussed above, jumping represents an important locomotion function for many biological organisms (e.g. locusts, fleas and jumping spiders) to extend navigation range and overcome obstacles, which also plays a crucial role in 3D soft robotics (Fig. 6I). Emulating the jumping behaviors of trap-jaw ants induced by the sudden snapping of their mandibles, Fig. 6J shows an insect-scale robot affording extraordinary jumping capability [84]. When one side of the Y-hinge is opened to an angle above 180° by a pair of spring actuators, a snap-through would occur to start one jump of the robot. Based on a soft electro-hydrostatic bending actuator, another soft jumping robot was fabricated, featuring rapid and continuous jumping and adjustable jumping direction (Fig. 6K) [85]. When applying a high electrical voltage on the actuators, the plastic ring frame would bend due to the dielectric liquid being squeezed into the air chamber by Maxwell stress, thus enabling a jump of up to 7.68 body heights with a forward speed of 6.01 BLs/second.

### 3D aquatic soft robotics

**Figure 7.**
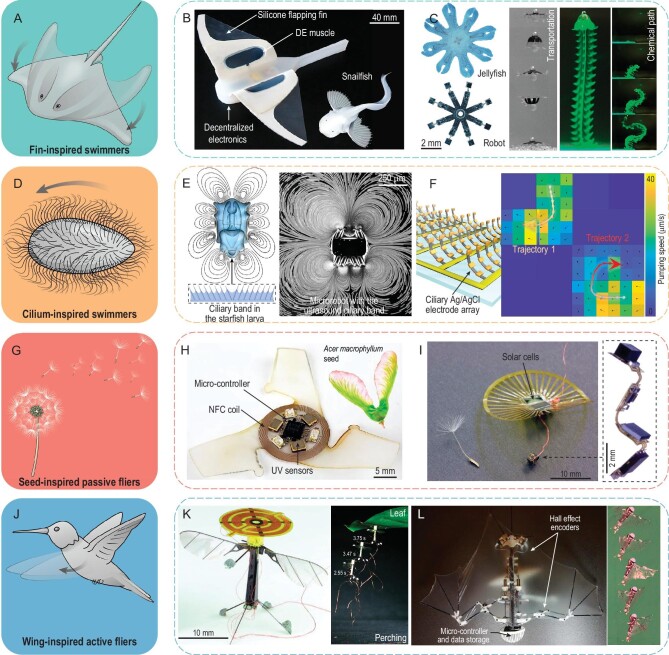
Bioinspired 3D soft aquatic and aerial robotics. (A) Illustration of fin-inspired 3D soft swimmers. (B) A snailfish-inspired deep-sea soft robot. (C) A jellyfish-like swimmer (left) capable of object transportation and chemical path generation (right). (D) Illustration of cilium-inspired 3D soft swimmers. (E) A starfish-like microswimmer with ultrasound-activated ciliary bands. (F) Electronic cilia array based on surface electrochemical actuators (left) capable of programmable microfluidic manipulation (right). (G) Illustration of seed-inspired passive fliers. (H) An *Acer macrophyllum* seed-inspired electronic flier. (I) A dandelion seed-inspired electronic flier. (J) Illustration of wing-inspired active fliers. (K) A flying insect-inspired aerial robot (left) capable of perching and takeoff using switchable electrostatic adhesion (right). (L) A bat-like robot with computing, sensing, and power electronics (left), featuring self-sustained flight (right). Adapted with permission from: (B) Ref. [86]. Copyright 2021 Springer Nature. (C) Ref. [88]. Copyright 2019 Springer Nature. (E) Ref. [89]. Copyright 2021 Springer Nature. (F) Ref. [90]. Copyright 2022 Springer Nature. (H) Ref. [91]. Copyright 2021 Springer Nature. (I) Ref. [92]. Copyright 2022 Springer Nature. (K) Ref. [94]. Copyright 2016 AAAS. (L) Ref. [95]. Copyright 2017 Springer Nature.

Fin-based flapping is the most widely adopted actuation mode for the aquatic swimming of bony fishes, which inspires the development of fish-like soft swimmers (Fig. 7A). For example, inspired by deep-sea snailfishes, an untethered soft robot with DE-based flapping fins was fabricated for deep-sea exploration (Fig. 7B). Note that onboard electronic circuits were detached and distanced to withstand the ultrahigh pressure in the Mariana Trench down to a depth of 10 900 meters [86]. A butterfly stroke-like soft swimmer actuated by a bistable actuator with two air channels was also realized [87], which can switch quickly between two stable states through elastic snap-through behaviors, thus enabling a record-high speed of 3.74 BLs/second and high maneuverability with a high turning speed of 157°/second. Inspired by the remarkable capabilities in fluidic flow control of jellyfishes, Fig. 7C shows an untethered swimmer consisting of eight magnetic elastomer lappets, which can transport objects and generate desired chemical paths in moderate Reynolds numbers with an oscillating magnetic field [88].

Microscale cilia broadly exist in diverse natural organisms and are capable of programmable nonreciprocal motions and inducing notable net fluid flows at very low Reynolds numbers (∼0.001 to 0.01) (Fig. 7D). Inspired by the natural ciliary systems, artificial cilia have been introduced into 3D soft swimmers for propulsion, liquid pumping/mixing and particle manipulation. For example, Fig. 7E shows an ultrasound-actuated microrobot consisting of synthetic ciliary bands, which is similar to the natural ciliary bands in the starfish larva [89]. By leveraging nonlinear acoustics, the ciliary bands undergo small amplitude oscillations and generate bulk fluid motions akin to a flow source or flow sink. Large deformations and manipulations of artificial cilia with a microlength of ∼50 μm become possible based on the surface electrochemical actuators made of layered platinum and titanium films (Fig. 7F) [90]. An active aquatic metasurface was demonstrated with an array of such cilia, which is capable of generating arbitrary and switchable microfluidic flow patterns.

### 3D aerial soft robotics

Bioinspired 3D soft fliers can be categorized into passive fliers (Fig. 7G–I) and active fliers (Fig. 7J–L) depending on the means of energy supply. Inspired by the passive falling behaviors of wind-dispersed seeds, a 3D electronic flier incorporating micro-controllers, near-field–communication coils, LEDs and ultraviolet sensors was realized based on the mechanically guided assembly (Fig. 7H) [91]. Featuring controlled rotational kinematics and low terminal velocities, the 3D flier can measure fine dust pollutions through the light dosimetry method. Based on similar biomimetic principles, a dandelion seed–inspired and battery-free flier is shown in Fig. 7I, which is powered by lightweight solar cells and can establish a backscatter communication link for data transmission [92]. Outdoor flying tests demonstrated a terminal velocity of ∼0.87 meters/second, 30 to ∼100 meters traveling distance in a gentle to moderate breeze at a 22-meter disposal height. On-demand shape-morphing capability can also be achieved by introducing active materials into 3D bioinspired passive fliers. A seed-inspired, throwable 3D electronic mesoflier was designed to realize on-demand fast unfolding (∼1 second) in the flight based on four electrothermal triggered SMP actuators [93].

Self-contained active flying of bioinspired 3D fliers inspired by wings improves the adaptivity to the surrounding environment for more complex tasks. Figure 7K shows a tethered insect-scale flapping wing robot capable of bionic perching and takeoff on overhangs based on a switchable electrostatic adhesion [94]. The interdigitated circular electrodes are integrated with the top of this robot using a polyurethane foam mount to induce sufficient electrostatic force between the electrodes and opposing surfaces for reliable perching on different surfaces, including glass, wood and a natural leaf. Bats with unrivaled agility and maneuvering characteristics inspired novel designs of a fully self-contained flying robot, which mimics the conformation of bat wings with active and passive joints (Fig. 7L) [95]. Based on a series of virtual constraints controlling the morphing wings, a number of autonomous flight maneuvers, including the zero-path flight, banking turn and diving, were demonstrated experimentally.

3D soft robotics capable of locomotion across different media (e.g. terrestrial/aquatic/aerial environments) represent another important and promising research direction, which would greatly enhance and expand the cross-medium operations of existing human-made rigid systems. Driven by predation, reproduction and other biological processes/consciousness, some animals have evolved the extraordinary ability of cross-medium locomotion. For example, frogs, salamanders and turtles are capable of crawling on land and swimming in water, flying fishes can glide short distances above the sea surface to avoid predators using their broad pectoral fins, and waterfowl (e.g. ducks, geese and swans) switch smoothly between walking, diving and flying. Such cross-medium locomotion modes provide useful guidelines for the design of 3D soft amphibious robotics, which typically involve the strategic integration of multiple independent actuation systems for different media or ingenious reconfiguration of a single actuation system during transitions of the medium boundary. For instance, an aerial-aquatic hitchhiking robot was developed by integrating bionic suckers and morphable propellers, which can fly, swim and attach to surfaces in both air and water [27]. Inspired by the morphology of a remora disc, the soft artificial sucker with separate lamellar compartments enables stable hitchhike even with partial adhesion. The reconfigurable propeller is capable of unfolding and folding passively in the air and water, separately, allowing the amphibious robot to cross the air-water boundary in 0.35 seconds.

## BIOINSPIRED 3D BIOMEDICAL DEVICES

Due to the intrinsically soft, curvy and high sensitivity to ambient environment change of tissues and organs in the human body, a number of variables should be taken into consideration when designing 3D biomedical devices, such as coordinated 3D shapes and mechanical stiffnesses with target tissues/organs, low biotoxic materials and controllable locomotion in luminal organs with confined spaces. The inspiration from natural organisms could play an important role in guiding the customization of device functions for different uses in the human body, especially for implantable applications (e.g. brain-computer interfaces, *in vivo* drug delivery and artificial prostheses).

**Figure 8.**
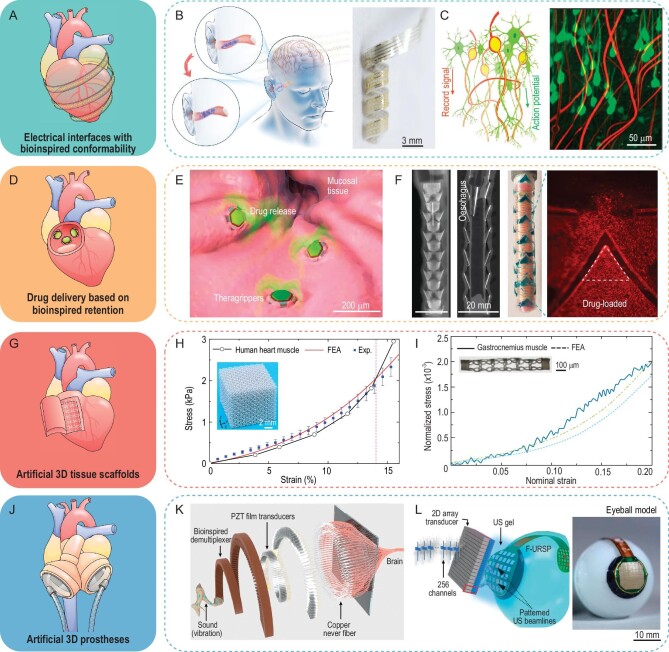
Bioinspired 3D biomedical devices. (A) Illustration of 3D electrical interfaces with bioinspired conformable electrodes. (B) Self-adapted helical in-ear electrodes used as brain-computer interfaces. (C) A neuron-inspired electrode network capable of recording action potentials. (D) Illustration of drug delivery based on bioinspired retention. (E) A hookworm teeth-inspired microgripper capable of controlled drug delivery in the gastrointestinal tract. (F) A scale-inspired kirigami stent capable of sustained drug delivery in luminal organs with pneumatic deployment. (G) Illustration of artificial 3D tissue scaffolds. (H) A bioinspired 3D lattice scaffold capable of reproducing nonlinear mechanical responses of the cardiac muscles. (I) A bioinspired tubular scaffold capable of reproducing nonlinear mechanical responses of the rat gastrocnemius muscles. (J) Illustration of artificial 3D prostheses. (K) Illustration of a cochlea-inspired artificial hearing system. (L) An artificial vision system capable of ultrasound-induced retinal stimulation. Adapted with permission from: (B) Ref. [13]. Copyright 2023 Springer Nature. (C) Ref. [96]. Copyright 2019 Springer Nature. (E) Ref. [18]. Copyright 2020 AAAS. (F) Ref. [98]. Copyright 2021 Springer Nature. (H) Ref. [100]. Copyright 2020 Springer Nature. (I) Ref. [101]. Copyright 2023 AAAS. (K) Ref. [102]. Copyright 2023 Wiley-VCH GmbH. (L) Ref. [16]. Copyright 2022 Springer Nature.

3D electrode-based biointerfaces typically rely on high-density electrodes distributed on 3D mesostructures/networks to collect spatiotemporal electrical signals or stimulate target cells, tissues and organs (Fig. 8A). Recently, a conformal in-ear flexible electrode inspired by the twining plants was fabricated for visual and auditory brain-computer interfaces (Fig. 8B) [13]. Under controlled electrothermal actuation of SMP layers, the bioelectrode can adaptively expand with a unique helical layout along the auditory meatus, ensuring a conformal contact and high-accuracy measurement of a brain electroencephalogram. Figure 8C shows an open 3D electronic network mimicking the subcellular structural features of neurons, consisting of an array of platinum recording electrodes [96]. Experimental results show that intimate interpenetration of the electronic network and neurons can be formed shortly following *in vivo* implantation.

Effective *in vivo* drug delivery has been used to cure various diseases, ranging from autoimmune disorders to cancer and bacterial infections, which can be realized by drug-delivery micro-robotics or microneedles with bioinspired retention mechanisms (Fig. 8D). The rapid development of 3D bioinspired microgrippers enables a number of new ways for *in vivo* drug delivery. Figure 8E shows a self-latching theragripper inspired by the ventricular teeth of the hookworm, which is capable of residing within the gastrointestinal tract for 24 hours by autonomously latching onto the mucosal tissue [18]. Microneedles-based drug delivery is one of the promising transdermal drug delivery methods, which can administer drugs in a painless manner for a long period of time. For example, a snake fang–inspired microneedle patch was realized, and the multiple open groove architectures in biomimetic microneedles enable the rapid and efficient delivery of liquid drugs/vaccines using the capillary effect under a gentle level of thumb pressure [97]. By adopting the snakeskin-inspired microneedles in a tubular pneumatic actuator, a deployable drug delivery system was shown in Fig. 8F, featuring sustainable and local release of drugs in luminal organs (e.g. the trachea, oesophagus and iliac artery) [98].

Well-designed prostheses of human tissues and organs should fit the anatomy structure, match the mechanical properties of surrounding tissues, and perform similar biological functions (Fig. 8G). For example, a heart valve scaffold was fabricated through a digital additive manufacturing method based on biocompatible silicone material, which can be applied for *in vivo* disease modeling and physical simulation [99]. Most biological tissues (e.g. cardiac muscles in the mammalian heart and human skin) would undergo nonlinear, anisotropic deformations under mechanical loadings and exhibit J-shape stress-strain curves. To capture such nonlinear mechanical responses of cardiac muscles, a rational design strategy of a bio-mimetic 3D network scaffold consisting of periodically arranged helical microstructures is presented in Fig. 8H [100]. The J-shaped stress-strain curves of cardiac muscles can be well emulated by tailoring the geometric parameters of helical microstructures. Similarly, a 3D tubular tissue scaffold with horseshoe-shape microstructures can be designed and fabricated to emulate the nonlinear mechanical responses of the rat gastrocnemius muscles (Fig. 8I) [101]. Note that current work on 3D bionic scaffolds mainly focus on the emulation of nonlinear mechanical responses of real tissues, and the scaffold design that takes into account the 3D vessels, nerves and mechanical properties has not yet been reported. Such a tissue scaffold with comprehensive bionic properties would be of great significance in medical practice, and promising approaches are emerging with the rapid development of advanced 3D manufacturing technologies. For example, based on the broad material compatibility of mechanically guided 3D assembly methods, 3D electronic scaffolds integrating soluble polymer materials (e.g. polyvinyl alcohol, chitosan and hydrolytic polyacrylamide) and conductive/semiconductive electronic materials (e.g. Au, Pt and Si) could be endowed with biomimetic geometries. 3D vascular microchannels could be formed after the solution of polymer materials. 3D interconnects with distributed electrodes allow the construction of bionic nerves, and their nonlinear mechanical properties could also be well-tailored based on rational optimizations.

By introducing electronic components into artificial 3D prostheses, multifunctional electronic prosthesis systems can be constructed with biomimetic pathways of electrical signals (Fig. 8J). For example, a biomimetic auditory system was realized based on a bioinspired demultiplexer, PZT film transducers and copper nerve fibers (Fig. 8K) [102]. A fine frequency resolution (up to ∼30 Hz) and a wide audible range (150 Hz to ∼12 000 Hz) were demonstrated experimentally. Figure 8L shows an electronic visual prosthesis integrating a 2D piezoelectric array, rectifiers, and a 3D stimulating electrode array with 32 pixels in a flexible printed circuit board, which is capable of providing prosthetic vision to people with acquired blindness [16]. Ultrasonic waves emitted by a transducer can be collected by the 2D piezoelectric array and then converted to electrical signals to initiate a neural response (i.e. the action potential) in cell populations of the retina.

## CONCLUSION AND OUTLOOK

Inspired by the long-term evolution of materials, structures, and locomotion modes in natural organisms, 3D bioinspired flexible devices and systems are rapidly advancing along three primary directions, including miniaturization, the increased merging of physical and artificial intelligence, as well as the enhanced adaptability and capabilities of heterogenous integration. In this review, we have highlighted the latest progress in bioinspired 3D flexible devices/systems, with a focus on the design concepts, manufacturing methods, structure-function principles and promising applications. We elaborated a list of representative 3D flexible devices/systems (e.g. 3D sensors, energy harvesters, optoelectronics, soft robotics and biomedical devices), with in-depth discussions on the bioinspired concepts, underlying relationships between devices’ function and 3D biological structures, and key performance metrics of these devices/systems.

**Figure 9.**
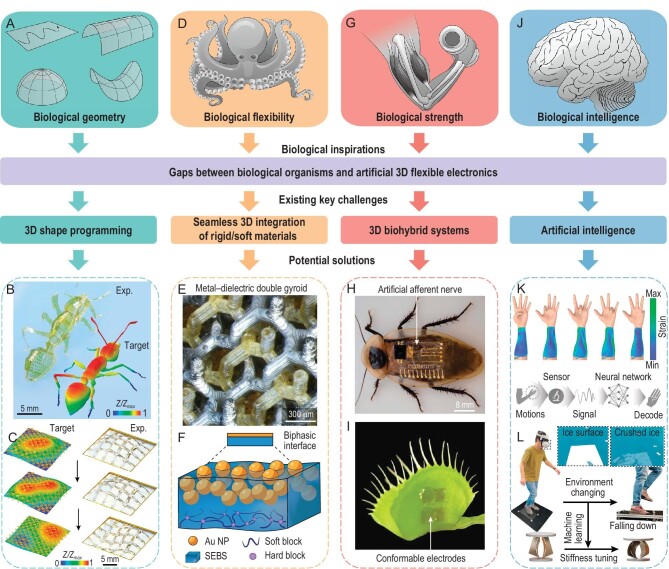
Key challenges existing in the design and fabrication of bioinspired 3D flexible electronics and potential solutions. (A) Illustration of diverse 3D biological shapes and the challenge in 3D shape programming. (B) 3D shape programming of an ant-like mesostructure based on the bioinspired microlattice design. (C) Dynamic programming of 3D mechanical metasurfaces actuated by distributed Lorentz forces. (D) Illustration of softness and flexibility of 3D biological materials and structures, and the challenge in seamless 3D integration of rigid/soft materials. (E) Programmed electrostatic charge deposition of electronic materials on the complex 3D double gyroid. (F) Illustration of a biphasic, nano-dispersed interface capable of providing continuous mechanical and electrical connections between different electronic modules. (G) Illustration of biological actuation forces (i.e. biological strength) and the challenge in realizing 3D biohybrid systems. (H) A cockroach integrated with a pressure sensor and an artificial afferent nerve on its back. (I) A biohybrid gripper with flexible electrodes attached to the flytrap epidermis. (J) Illustration of biological intelligence and the challenge in the development of artificial intelligence. (K) A skin-integrated strain sensor capable of measuring hand skin deformations and decoding the 3D finger gestures based on a deep neural network. (L) Human-machine interactions based on 3D bimodal, stiffness-tuning origami structures and machine learning algorithms. Adapted with permission from: (B) Ref. [103]. Copyright 2023 AAAS. (C) Ref. [104]. Copyright 2022 Springer Nature. (E) Ref. [107]. Copyright 2020 Springer Nature. (F) Ref. [108]. Copyright 2023 Springer Nature. (H) Ref. [109]. Copyright 2018 AAAS. (I) Ref. [110]. Copyright 2021 Springer Nature. (K) Ref. [111]. Copyright 2020 Springer Nature. (L) Ref. [12]. Copyright 2023 Springer Nature.

Despite the remarkable progress, many scientific and technological challenges still remain, due to the great gap between natural organisms and artificial electronic devices, such as the 3D shape programming of bioinspired structures/devices with complex topologies (Fig. 9A–C), seamless 3D integration of electronic materials (e.g. metals and semiconductors) and soft materials (especially soft active materials) (Fig. 9D–F), effective synergy of electronics and cells/tissues for hybrid electronics (Fig. 9G–I), and the application of embedded artificial intelligence in 3D bioinspired devices (Fig. 9J–L). This section discusses potential solutions to these challenging issues and introduces a few exciting directions of 3D bioinspired flexible devices/systems for future exploration.

### 3D shape programming for precisely reproducing biological surfaces

Programming 3D shapes of biomimetic artificial structures at the microscale based on 2D-to-3D assembly is crucial for the development of novel electronic devices, especially for biomedical devices. The 3D shapes of biological cells/tissues/organs are closely tied to their physiological functions. Representative examples include the hemispherical retina for wide-field-of-view visual perception, the highly convoluted structure of the brain with gyri and sulci for the accommodation of a larger number of neurons, as well as the pear-like shape of the stomach ensuring the storage and controlled release of food into the small intestine. Precise shape programming allows the construction of 3D electronic devices and systems with the desired curvature distributions for either conforming to or replicating the curvy surfaces of biological tissues and organs, which is necessary for the monitoring or emulation of their physiological functions. However, the intricate mapping between 3D microstructures and 2D precursor patterns, and the nonlinear deformations during shape transformation pose great challenges in 3D shape programming, calling for rational design strategies and advanced prediction algorithms (Fig. 9A). Recently-developed rational assembly methods provide a possible solution to this problem [103]. The rational microlattice design inspired by cellular microstructures in natural organisms allows the transformation of 2D thin films into target 3D curved mesosurfaces through compressive buckling deformations. Analytical modeling and a machine learning–based computational approach enable the accurate prediction of porosity distribution and compressive strains for the assembly of target biological mesosurfaces (e.g. the ant-like mesosurface in Fig. 9B). Mimicking the retina, a spherical cap–shaped electronic cell scaffold with integrated sensing capabilities was fabricated based on microlattice designs, which is capable of studying real-time, spatial distributions of physiological activities (e.g. growth and apoptosis) of retinal pigment epithelium cells in a noninvasive manner. Dynamic 3D shape-morphing is ubiquitous in living organisms, while it is challenging to continuously reprogram target shapes after fabrication. Figure 9C presents a metasurface constructed from an array of serpentine interconnects with ultra-low rigidity, featuring dynamic replication of 3D complex shapes under the actuation of distributed Lorentz forces [104]. Note that the microlattice design and reprogrammable metasurface design both find limitations in realizable 3D geometries (e.g. 3D hierarchical structures with high-order micropatterns), due to the bending-dominated global deformation characteristics and the coordination between adjacent ribbons, respectively. Therefore, a generic shape programming method affording nearly arbitrary 3D geometries and broad material applicability still needs to be explored for bioinspired 3D flexible devices and functional systems.

### Seamless 3D rigid/soft integration for bioinspired flexible electronics

Soft active materials, widely used in bioinspired 3D flexible devices/systems for robotic actuation and biomedical engineering, is intrinsically incompatible with most inorganic electronic materials (e.g. metals, semiconductors and piezoelectric ceramics) (Fig. 9D). For example, soft actuators with integrated actuation/sensing capabilities are of great significance for the increase in degree of integration and the reduction of feature size of 3D soft robotics. Typically, actuation strains of soft active materials (e.g. LCE) are above 30%, while the plastic strains of metals are below 1% and the fracture strain of monocrystalline silicon is ∼2%. Such an insurmountable gap could easily lead to interfacial failure (e.g. delamination, fracture and electromigration), in particular for implantable long-term devices, where durability is a crucial requirement.

Patterning electronic materials with stretchable layouts (e.g. serpentine, fractal and helical geometries), followed by transfer printing onto soft active materials, represents a widely used strategy at the current stage of development, while interfacial detachment and significant local strain concentration could occur at the interfaces of 3D rigid/soft materials [105,106]. Possible solutions to seamless 3D rigid/soft integration for bioinspired flexible devices could lie in developing new 3D multi-material printing technologies and exploring new integration schemes for inorganic electronic materials and soft materials. Recently, a charge-programmed 3D printing technology was developed, allowing selective volumetric depositions of metals, magnetic materials and colloidal materials (Fig. 9E) [107]. 3D printed complex microstructures with a programmed surface charge mosaic were taken as a deposition platform of functional materials based on localized electrostatic attraction. Explorations of new integration schemes focused on the surface modification of liquid alloys and designs of metal-polymer hybrid interfaces. For example, by thermally evaporating Au or Ag nanoparticles onto a styrene-ethylene-butylene-styrene (SEBS) thermoplastic elastomer, interpenetrating nanostructures can be formed (Fig. 9F), providing both continuous mechanical and electrical pathways [108]. Based on this technology, biphasic interfaces could be realized and used to connect soft electrodes, rigid circuit board and encapsulation layers in a plug-and-play way without any pastes.

### Biohybrid electronic systems with enhanced physical intelligence

Despite enormous efforts on imparting biological functions into artificial devices and systems based on engineering materials, it is still almost unattainable to fully replicate the peculiarity, strength and intelligence of living organisms (Fig. 9G). Merging living organisms with flexible electronics allows the development of biohybrid electronic systems with the physical intelligence (e.g. autonomous energy generation, high power-to-weight ratios, real-time reactions to changing environments and self-healing) of natural organisms. For example, by incorporating an artificial afferent nerve to the motor nerves of a Discoid cockroach, a hybrid bioelectronic reflex arc can be realized to actuate muscles at the leg joint (Fig. 9H) [109]. Similarly, Fig. 9I presents an electrical plant-hybrid actuator with conformal electrodes integrated on the surface of the leaves of a Venus flytrap [110]. Based on the frequency-dependent action-potential modulation, the leaves can be actuated to open and close reversibly with a very low power input (∼10^−5^ W). Till now, actuation modes of biohybrid devices are still very simple, mainly restricted to unidirectional bending, contraction, opening and closing. Open opportunities may lie in exploring new 3D integration schemes of cell/tissue/organs and electronic components with more stimulating points (e.g. increased electrode resolution), and new coordinated control strategies to access more complex actuation modes. Due to the involvement of living components, the long-time stability and self-healing property of biohybrid electronics deserve further investigation.

### Embedded artificial intelligence for autonomous 3D electronic systems

Bioinspired designs adopted in 3D flexible sensors allow high sensitivity and broad sensing range of diverse physical parameters in the ambient environment, which could be used to accumulate a big dataset. By introducing artificial intelligence (AI) in the signal processing flow, the underlying valid information could be intelligently filtered from high-throughput big data, and then an autonomous system could be realized. For example, based on measured real-time distributions of the liquid pressure throughout the body of a fish-like robotic swimmer, high-resolution characteristics of ambient flow (e.g. flow velocity, water depth and vortex structures), and vibration signals from a predator could be instantly identified with the aid of AI. Then, the most appropriate coping strategy (e.g. avoidance, acceleration or following) can be selected based on the AI judging algorithm. Such AI-based sensing systems have been initially elucidated with planar flexible sensors. For example, an e-skin integrated with a deep neural network is shown in Fig. 9K, which is capable of decoding dynamic motions of five fingers in real-time from a distance, without the need to use a large-area sensor array [111]. Note that the rapid situation learning algorithm was adopted to ensure stable operation regardless of the wrist position. Since the existing efforts mainly focused on gesture recognition and object sorting based on planar flexible sensors, future studies could follow by extending the AI technology to bioinspired 3D flexible sensors/robotics in order to complete much more complex tasks (e.g. intelligent perception and autonomous locomotion of soft robotics based on AI and human-machine interactions). For example, human-machine interactions have been demonstrated based on 3D sensors/actuators and AI algorithms (Fig. 9L), which can be used in prosthetic control/feedback and simulating movement (e.g. walking on thin ice or grass in the virtual environment) [12].

Development of new bioinspired 3D flexible devices and functional systems requires coordinated innovations in bioinspired principles, composing materials, structural designs and manufacturing methods. With critical challenges addressed, 3D flexible devices/systems with diverse and extraordinary functionalities would meet ever-growing demands in intelligent sensing, health monitoring and human-machine interactions.

## Supplementary Material

nwad314_Supplemental_File
