# Supplementary material for: Bioinspired 3D flexible devices and functional systems
Source: Natl Sci Rev. 2023 Dec 13;11(3):nwad314. doi: 10.1093/nsr/nwad314 (PMC10833470; doi:10.1093/nsr/nwad314)
Supplement: nwad314_Supplemental_File [file nwad314_supplemental_file.pdf]

Supplementary Information for

Bioinspired 3D flexible devices and functional systems

Xu Cheng[1, 2, †], Zhangming Shen[1, 2, †] and Yihui Zhang[1, 2, *]

[1]Applied Mechanics Laboratory, Department of Engineering Mechanics, Tsinghua University, Beijing 100084, China;

[2]Laboratory of Flexible Electronics Technology, Tsinghua University, Beijing 100084, China
[*]**Corresponding author.** E-mail: yihuizhang@tsinghua.edu.cn
[†]Equally contributed to this work.

Table S1. Representative manufacture methodologies of bioinspired 3D flexible devices/systems

Categories	Methods	Techniques	Materials	Advantages	Limitations	Applications
**Direct 3D manufact- -ure**	3D printing [1]	DIW, FDM, DLP, TPL, LAPμSL	Photocuring resin, liquid metal, viscoelastic ink	Wide feature sizes, arbitrary 3D shapes, multi-materials	Restricted material types, relatively low speed	Sensors [2], robots [3], prostheses [4]
	Laser ablation [5]	Laser engraving, cutting and tuning	Laser/heat-absorbable materials	Broad material types, wide feature sizes	Limited 3D shapes, heat-induced material damage	Sensors [6], robots [7], cameras [8]
	Templating [9]	Casting, coating, soft lithography	Curable polymers, viscous materials	Wide feature sizes, low requirements on machines	Restricted to polymer materials, limited shapes	Sensors [10], robots [11], cameras [12]
**2D-to-3D assembly**	Swelling actuation [13]	4D printing, programmed polymerization	Hydrogels, hydrogel-filler composites	Wide feature sizes, high geometric complexity, programmable morphing	Restricted to polymer materials, low accuracy, limited speed	Robotics [14], energy harvesters [15]
	Thermal actuation [16]	4D printing, programmed heating	Materials with heat-induced volume/phase changes	Wide feature sizes, high geometric complexity, programmable morphing	Restricted material types, low accuracy, relatively high temperature	Robotics [17], biomedical devices [18]
	Light actuation [19]	4D printing, programmed lighting	Polymers with light-absorbing nanoparticles or photoactive groups	Wide feature sizes, remote activation, easy manipulation at small scales	Restricted material types, limited light wavelengths and intensity	Energy harvesters [20], robots [21]
	Electroma- -gnetic actuation [22]	4D printing, programmed magnetic field	Polymers with embedded ferromagnetic particles, flexible wires	Wide feature sizes, high geometric complexity, fast shape morphing	Restricted to magnetic materials, strong magnetic fields, complex systems	Robotics [23], biomedical devices [24]
	Pneumatic actuation [25]	3D printing, programmed air pressure	Polymers embedded with microchannels	Fast and reversible 3D shape morphing, high actuation force and shape complexity	Restricted to polymers, tethered configurations, complex gas system	Robots [26], biomedical devices [27], cameras [28]
	Mechanical- -ly guided buckling [29]	Transfer printing, strategically designed 2D precursors and substrates	Polymers, metals, semiconductors, piezoelectric materials, 2D materials	Broad materials applicability, high geometric complexity, wide feature sizes and excellent compatibility with planar microfabrication	Constraints of supporting elastomeric substrates	Sensors [30], energy harvesters [31], robots [32], biomedical devices [33]

References in Supplementary Information

1. Wallin TJ, Pikul J and Shepherd RF. 3D printing of soft robotic systems. *Nat Rev Mater* 2018; **3**: 84-100.
2. Cheng W, Wang X, Xiong Z *et al.* Frictionless multiphasic interface for near-ideal aero-elastic pressure sensing. *Nat Mater* 2023; **22**: 1352–60.
3. Zeng H, Wasylczyk P, Parmeggiani C *et al.* Light-Fueled Microscopic Walkers. *Adv Mater* 2015; **27**: 3883-7.
4. Coulter FB, Schaffner M, Faber JA *et al.* Bioinspired Heart Valve Prosthesis Made by Silicone Additive Manufacturing. *Matter* 2019; **1**: 266-79.
5. Zeng H, Du XW, Singh SC *et al.* Nanomaterials via Laser Ablation/Irradiation in Liquid: A Review. *Adv Funct Mater* 2012; **22**: 1333-53.
6. Miao LM, Song Y, Ren ZY *et al.* 3D Temporary-Magnetized Soft Robotic Structures for Enhanced Energy Harvesting. *Adv Mater* 2021; **33**: 2102691.
7. Iyer V, Gaensbauer H, Daniel TL *et al.* Wind dispersal of battery-free wireless devices. *Nature* 2022; **603**: 427-33.
8. Wu D, Wang J-N, Niu L-G *et al.* Bioinspired Fabrication of High-Quality 3D Artificial Compound Eyes by Voxel-Modulation Femtosecond Laser Writing for Distortion-Free Wide-Field-of-View Imaging. *Adv Opt Mater* 2014; **2**: 751-8.
9. Rogers JA and Nuzzo RG. Recent progress in soft lithography. *Mater Today* 2005; **8**: 50-6.
10. Liu L, Niu S, Zhang J *et al.* Bioinspired, Omnidirectional, and Hypersensitive Flexible Strain Sensors. *Adv Mater* 2022; **34**: e2200823.
11. Ren Z, Hu W, Dong X *et al.* Multi-functional soft-bodied jellyfish-like swimming. *Nat Commun* 2019; **10**: 2703.
12. Dai B, Zhang L, Zhao C *et al.* Biomimetic apposition compound eye fabricated using microfluidic-assisted 3D printing. *Nat Commun* 2021; **12**: 6458.
13. Ionov L. Biomimetic Hydrogel‐Based Actuating Systems. *Adv Funct Mater* 2013; **23**: 4555-70.
14. Zhang Y, Zhang F, Yan Z *et al.* Printing, folding and assembly methods for forming 3D mesostructures in advanced materials. *Nat Rev Mater* 2017; **2**: 17019.
15. Cezan SD, Baytekin HT and Baytekin B. Self-Regulating Plant Robots: Bioinspired Heliotropism and Nyctinasty. *Soft Robot* 2020; **7**: 444-50.
16. Karothu DP, Mahmoud Halabi J, Li L *et al.* Global Performance Indices for Dynamic Crystals as Organic Thermal Actuators. *Adv Mater* 2020; **32**: e1906216.
17. Pang W, Xu S, Wu J *et al.* A soft microrobot with highly deformable 3D actuators for climbing and transitioning complex surfaces. *Proc Natl Acad Sci USA* 2022; **119**: e2215028119.
18. Wang Z, Shi N, Zhang Y *et al.* Conformal in-ear bioelectronics for visual and auditory brain-computer interfaces. *Nat Commun* 2023; **14**: 4213.
19. Stoychev G, Kirillova A and Ionov L. Light‐Responsive Shape‐Changing Polymers. *Adv Opt Mater* 2019; **7**: 1900067.
20. Qian X, Zhao Y, Alsaid Y *et al.* Artificial phototropism for omnidirectional tracking and harvesting of light. *Nat Nanotechnol* 2019; **14**: 1048-55.
21. Miskin MZ, Cortese AJ, Dorsey K *et al.* Electronically integrated, mass-manufactured, microscopic robots. *Nature* 2020; **584**: 557-61.
22. Yunas J, Mulyanti B, Hamidah I *et al.* Polymer-Based MEMS Electromagnetic Actuator for Biomedical Application: A Review. *Polymers* 2020; **12**: 1184.
23. Soon RH, Yin Z, Dogan MA *et al.* Pangolin-inspired untethered magnetic robot for on-demand biomedical heating applications. *Nat Commun* 2023; **14**: 3320.
24. Ghosh A, Li L, Xu L *et al.* Gastrointestinal-resident, shape-changing microdevices extend drug release in vivo. *Sci Adv* 2020; **6**: eabb4133.
25. Xavier MS, Tawk CD, Zolfagharian A *et al.* Soft Pneumatic Actuators: A Review of Design, Fabrication, Modeling, Sensing, Control and Applications. *IEEE Access* 2022; **10**: 59442-85.
26. Chi YD, Hong YY, Zhao Y *et al.* Snapping for high-speed and high-efficient butterfly stroke-like soft swimmer. *Sci Adv* 2022; **8**: eadd3788.

27. Babaee S, Shi Y, Abbasalizadeh S *et al.* Kirigami-inspired stents for sustained local delivery of therapeutics. *Nat Mater* 2021; **20**: 1085-92.
28. Jung I, Xiao J, Malyarchuk V *et al.* Dynamically tunable hemispherical electronic eye camera system with adjustable zoom capability. *Proc Natl Acad Sci USA* 2011; **108**: 1788-93.
29. Bo R, Xu S, Yang Y *et al.* Mechanically-Guided 3D Assembly for Architected Flexible Electronics. *Chem Rev* 2023; **123**: 11137–89.
30. Cheng X, Liu Z, Jin T *et al.* Bioinspired design and assembly of a multilayer cage-shaped sensor capable of multistage load bearing and collapse prevention. *Nanotechnology* 2021; **32**: 155506.
31. Han M, Wang H, Yang Y *et al.* Three-dimensional piezoelectric polymer microsystems for vibrational energy harvesting, robotic interfaces and biomedical implants. *Nat Electron* 2019; **2**: 26-35.
32. Kim BH, Li K, Kim JT *et al.* Three-dimensional electronic microfliers inspired by wind-dispersed seeds. *Nature* 2021; **597**: 503.
33. Cheng X and Zhang Y. Micro/Nanoscale 3D Assembly by Rolling, Folding, Curving, and Buckling Approaches. *Adv Mater* 2019; **31**: e1901895.
